# *Shigella*-associated mild encephalitis with reversible splenial lesion in Hospital Center Delafontaine, Saint-Denis, France: a case report

**DOI:** 10.1186/s12887-022-03460-6

**Published:** 2022-07-15

**Authors:** Louise Le Soudéer, Jeanne Truong, Julie Le Gal, Simon Escoda

**Affiliations:** Service de Pédiatrie, Centre Hospitalier Delafontaine, 93200 Saint-Denis, France

**Keywords:** Case report, Paediatric, *Shigella*, MERS

## Abstract

**Background:**

Mild encephalitis with reversible splenial lesion of the corpus callosum (MERS) is a clinical/radiological syndrome characterized by hyperintense signal changes in the splenium of the corpus callosum visible on diffusion weighted imaging (DWI) in the brain Magnetic Resonance Imaging (MRI) associated with various neurological symptoms. Progression is usually favorable with disappearance of the MRI brain lesion and regression of clinical symptoms over a few days to a few weeks. The exact pathophysiology remains unclear. MERS can be associated with various pathogens.

**Case presentation:**

We report here a paediatric case of MERS associated with *Shigella flexneri* infection. A five-year-old boy with no relevant past medical history presented with symptoms such as headache, fever, profuse diarrhea and hallucinations.

A brain Magnetic Resonance Imaging performed on Day 2 of the symptoms revealed hyperintense signal changes of the splenium of the corpus callosum in T2 FLAIR sequence. This infection had a favorable outcome after antibiotic therapy. No further recurrence of symptoms was observed and a follow-up clinical examination eight weeks later was normal. A follow-up brain Magnetic Resonance Imaging three months after discharge was also normal and the hyperintense signal changes of the splenium of the corpus callosum had disappeared completely.

**Conclusions:**

MERS is a clinical/radiological syndrome with a generally good prognosis.

We believe that this is the first description of a case of *Shigella*-associated MERS.

It is useful to know about this condition to help distinguish it from acute disseminated encephalomyelitis.

## Background

Mild encephalitis with reversible splenial lesion of the corpus callosum (MERS) is a clinical/radiological syndrome. It is not a rare condition in a paediatric setting. As an example two cases have been recorded in our hospital over an 18-month period (2019–2020).

We present here a case of MERS in a five-year-old boy associated with *Shigella flexneri* infection. We believe that this is the first description of a case of *Shigella*-associated MERS.

### Case presentation

A five-year-old boy with no relevant past medical history was admitted to Delafontaine Hospital Center, Saint-Denis, France, with insomnia-causing headache, fever and profuse diarrhea without mucous or blood over a 24-h period. He also presented with temporal and spatial disorientation, obtundation and hallucinations that started a few hours prior to Attendances and Emergency Department (A&E) admission. These symptoms lasted 24 h after temperature came back to normal. No specific contagion, no recent travel or contact with animals were reported. The vaccinations of the child were not up to date according to the French vaccination schedule.

When admitted to paediatric A&E, his vital signs were: temperature 38.8 °C, heart rate 140 beats/min, respiratory rate 30 breaths/min, oxygen saturation 98% at ambient air and blood pressure, 106/77 mmHg. He was confused, disoriented in time and space and suffering from visual hallucinations. The rest of the clinical examination, particularly neurological, was normal.

The blood count was normal. The serum electrolytes showed: a slight hyponatremia (serum sodium, 132 mmol/L) and an inflammatory syndrome: C-reactive protein, 118 mg/L and procalcitonin, 4.51 ng/mL. Liver function tests were normal. Blood cultures were negative after five days. Cerebrospinal fluid (CSF) analysis revealed: 1 leucocyte/mm^3^ per 6 red blood cells/mm^3^; protein concentration, 0.16 g/L and CSF versus blood glucose ratio > 0.6. The direct CSF examination was negative and cultures were sterile after five days. PCR for Herpes Simplex Virus 1 and 2, varicella and enterovirus in the CSF were negative. Stool virology (rotavirus, adenovirus) was negative while the stool culture identified the presence of *Shigella flexneri*.

Given the SARS-CoV-2 pandemic context, tests for Paediatric Inflammatory Multisystem Syndrome were performed, which included an echocardiography, troponin and NT-pro-BNP. The results all came back normal. Nasal PCR and SARS-CoV-2 serology were negative.

**Fig. 1:**
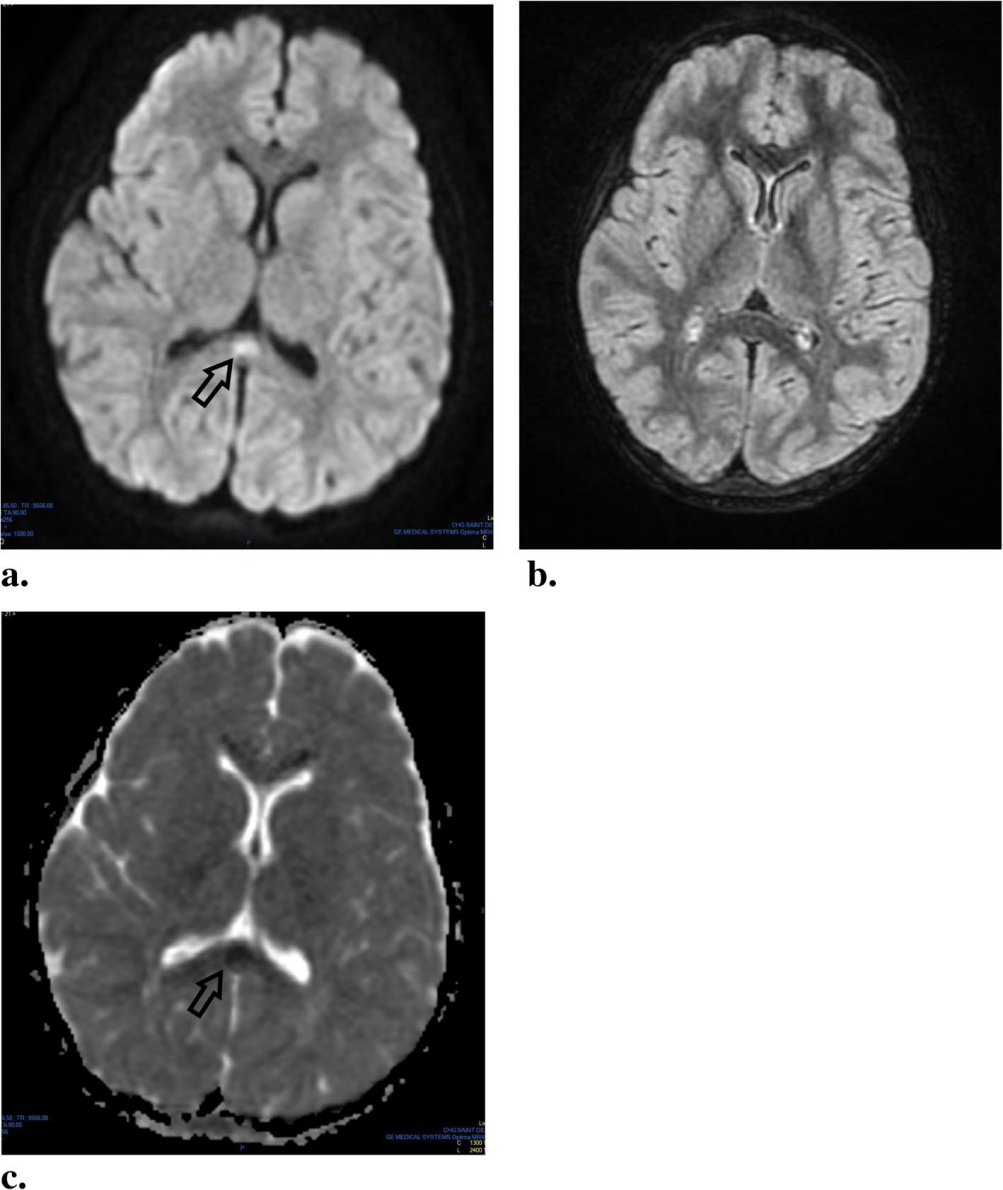
This is the legend from a figure from the study. **a.** Brain MRI performed on day 2 of the symptoms. Hyperintense signal changes of the SCC are visible on Diffusion-weighted imaging sequence. **b.** Brain MRI performed on day 2 of the symptoms. No hyperintense signal changes of the SCC are visible on T2W/FLAIR image. **c.** Brain MRI performed on day 2 of the symptoms. Reduction of ADC is visible on ADC maps

A non-enhanced brain scan (CT) showed no evidence of intracranial lesion. A brain MRI performed on Day 2 of the symptoms revealed hyperintense signal changes on diffusion weighted imaging (DWI) sequences of the splenium of the corpus callosum (SCC), and diffusion sequences with a restriction of the apparent diffusion coefficient (ADC) (Fig. 1). However in our case there were no hyperintense signal changes in T2 Fluid Attenuated Inversion Recovery (FLAIR).

The initial electroencephalogram (EEG) performed on Day 2 also revealed greater slow wave activity in the right frontal and temporal leads, consistent with encephalitis.

Given the encephalopathic symptoms, intravenous treatment with cefotaxime and acyclovir at meningeal doses was rapidly initiated on the day of admission.

Acyclovir was discontinued after receiving a negative CSF PCR for herpes simplex virus. The dose of cefotaxime was rapidly reduced to 100 mg/kg/d due to the negative direct CSF examination. Antibiotic treatment was continued for three days to treat the *Shigella* infection.

The inflammatory syndrome reduced during hospitalization, with C-reactive protein of 93 mg/L at Day 5. The clinical condition of the patient improved during hospitalization with complete regression of neurological symptoms. The condition of the patient was normal at Day 4 post-admission and he was discharged after six days in hospital.

No further recurrence of symptoms was observed and a follow-up clinical examination eight weeks later was normal.

A follow-up EEG four weeks after discharge was normal.

A follow-up brain MRI three months after discharge was also normal and the SCC hyperintense signal changes had disappeared completely.

## Discussion and conclusions

MERS is a clinical/radiological syndrome first described by Tada et al. In 2004 [1]. Its radiological presentation is established on the basis of a brain MRI: SCC hyperintense signal changes on T2 FLAIR and DWI sequences. The apparent diffusion coefficient (ADC) of the lesion is decreased on ADC maps, and iso- to hypo-intense signals may appear on T1-weighted imaging sequences without enhancement after injection of a contrast agent [1–3].

MERS has been widely described in case reports and case series around the world, initially first in Japan. The clinical symptoms of MERS described in the literature are varied and affect the central nervous system: disturbed consciousness, behavioral disorders, incoherent speech, seizures, drowsiness, headaches, hallucinations, and more rarely ataxia, motor deficits and blindness. There could be also more general symptoms such as fever, cough, vomiting and/or diarrhea.

No specific biological criteria defining MERS have been established. Pleocytosis and hyponatremia have been reported inconsistently [1, 2, 4]. Pleocytosis was not observed in our case: however, mild hyponatremia was reported and was rapidly corrected with the resolution of the symptoms. An inflammatory syndrome is often identified.

There is a frequent but not universal association between MERS and hyponatremia. The principal cause of hyponatremia mentioned so far is the syndrome of inappropriate antiduretic hormone secretion (SIADH). Interleukin 6 (IL-6) which increases during inflammation, may have a key role in the pathophysiology of SIADH. Indeed, it is recalled that it can stimulate the hypothalamus and pituitary gland and induce vasopressin release leading to hyponatremia [5, 6].

The possible etiologies for MERS are large. Historically, reversible MRI lesions in the SCC were described in patients using anti-epileptic drugs [7]. Anti-epileptic drugs and their rapid withdrawal or toxic levels were thought to be the cause of the splenial lesion although it is difficult to strongly impute the antiepileptic drugs over the effect of frequent seizures in epileptic patients [8]. The principal hypothesis is that antiepileptic drugs may interact with fluid balance system (arginine-vasopressin) and cation channels. If antiepileptic drugs concentration changes rapidly, this might disturb the fluid balance system, causing a syndrome of inappropriate antidiuresis that leads to hyponatremia and finally to brain edema. However this hypothesis cannot explain why the splenium of the corpus callosum is the preferential localization.

Reversible MRI lesions in the SCC have also been described in high-altitude disease, cerebrovascular disease or vasculitis (Kawasaki disease), metabolic disturbances (hypoglycemia, Machiafava-Bignami disease) [9] or infectious diseases.

The list of MERS-associated pathogens identified includes viruses such as rotavirus [1, 2], cytomegalovirus [10], adenovirus [11], influenza A/B virus [12], herpesvirus 6 [13] and Epstein Barr virus [14], as well as bacteria such as *Mycoplasma pneumonia* [4]*, Salmonella *[15]*, Listeria monocytogenes *[16]*, Escherichia coli *[17]*, Klebsiella pneumoniae *[18] and *Enterococcus faecalis *[19]*.* It is known that *Shigella-*associated infections are sometimes associated with neurological symptoms such as seizures, drowsiness, hallucinations and, more rarely, severe and fatal encephalopathy [20, 21]. However, the association with MERS had never been mentioned so far.

As described in our clinical case, most MERS patients recover within a few days with a favorable prognosis and the brain MRI usually reverts to normal within a month. However, a poor clinical outcome has been reported in some cases, without a clear explanation. In addition, in some of the cases described, the SCC lesion did not revert on a subsequent brain MRI after one month [22, 23]. Two types of radiological MERS have been described in the literature [24]. Type I is an isolated SCC lesion, whereas type II is described as an extensive lesion, encompassing the entire corpus callosum and sometimes the white matter. Although radiological lesions are usually reversible within a few weeks, some cases of Type II MERS have reported clinical neurological sequelae and radiological lesions persisting for over a month [4, 25].

At present, the exact pathophysiological mechanism of MERS is not fully understood. The most commonly described hypothesis is cytotoxic edema caused by cytokine release, as high density of cytokine receptors is observed in the SCC [3]. Indeed, it is well known that pro-inflammatory cytokines such as IL-1 and IL-6 are usually released due to endothelial damage. Theses cytokines lead to activate receptors such as N-methyl-D-aspartate (NMDA), aquaporines 4 (AQP4) at the surface of astrocytes and neurons. It could result in an influx of water into these cells (cellular swelling). As there is a higher density of cytokine and AQP4 receptors in the neurons, astrocytes and oligodendrocytes of the corpus callosum, this localisation is more likely to be exposed to cytotoxic edema when cytokine release occurs.

Cytotoxic edema is characterized by the intracellular accumulation of fluid and sodium, which results in cell swelling. It is generally known to lead to irreversible lesions and cellular death, such as cytotoxic edema in ischemic strokes. However, MRI shows a reversible lesion on diffusion-weighted imaging, separating these lesions from persistent ischemia [3].

The primary differential diagnosis of MERS appears to be acute disseminated encephalomyelitis (ADEM) [1]. ADEM is described as an inflammatory disorder usually occurring after infection or vaccination and characterized by multifocal demyelination of the central nervous system. Its clinical presentation may be similar to MERS: seizures, altered consciousness, focal neurological signs. Symptoms develop over a few days to weeks after the start of the suspected triggering element. Despite the similar presentation, ADEM can be severe and in some cases, including acute necrotizing encephalitis lead the children to death. MRI shows hyperintense asymmetric signal in T2 FLAIR sequences, most commonly in white matter but sometimes also in the thalami and basal ganglia. Areas of enhancement are described in T1-weighted images with gadolinium contrast. In acute stages the lesions may show diffusion restriction on diffusion-weighted sequences.

As seen in MERS, CSF pleocytosis is common in ADEM. Assessment of intratecal antibodies synthesis through oligoclonal bands can be also informative since there are some cases of ADEM that shows oligoclonal bands in the CSF [26].

Unfortunately we did not search for oligoclonal bands in the CSF of our patient.

Treatment of ADEM is via high-dose intravenous corticosteroids. Lesions may develop over weeks to months and only disappear after several months, although white-matter lesions can sometimes become permanent. Children commonly recover completely usually over 4–6 weeks but in some cases they may suffer from cognitive and/or physical deficits. Nevertheless long-term clinical and radiological follow-up of ADEM is important. Sometimes another episode of ADEM can occur more than 3 months following the initial condition and is referred to as multiphasic ADEM.

MERS is a clinical/radiological syndrome with a generally good prognosis. The pathophysiological mechanism is still unclear. It is useful to know about this condition to help distinguish it from ADEM. MERS can be associated with various pathogens. We report here a paediatric case of MERS associated with *Shigella flexneri* infection, which had a favorable outcome after antibiotic therapy. 

## Data Availability

The dataset supporting the conclusions of this article is included within the article.

## Abbreviations

ADC: Apparent diffusion coefficient
ADEM: Acute disseminated encephalomyelitis
A&E: Attendances and emergency department
CSF: Cerebrospinal fluid
CT-Scan: Computerized-tomography scan
DWI: Diffusion weighted imaging
EEG: Electroencephalogram
FLAIR: Fluid attenuated inversion recovery
IL-6: Interleukin-6
MERS: Mild encephalitis with a reversible splenial lesion of the corpus callosum
MRI: Magnetic resonance imaging
PCR: Polymerase chain reaction
SIADH: Syndrome of inappropriate antidiuretic hormone secretion
SCC: Splenium of the corpus callosum
